# Genome-wide identification of glyoxalase (*PbrGLY*) gene family and functional analysis of *PbrGLYI-28* in response to *Botryosphaeria dothidea* in pear

**DOI:** 10.1186/s12870-025-06302-6

**Published:** 2025-03-18

**Authors:** Fei Wang, Fengpei Sun, Zhaoyi Yu, Yue Zhang, Yuting Liu, Xiaolei Sun, Dan Li, Shaoling Zhang, Xun Sun

**Affiliations:** National Key Laboratory of Crop Genetics & Germplasm Enhancement and Utilization, College of Horticulture, Sanya Institute of Nanjing Agricultural University, Nanjing Agricultural University, Nanjing, Jiangsu 210095 China

**Keywords:** *Botryosphaeria dothidea*, Glyoxalase, Pear

## Abstract

**Background:**

Glyoxalase (GLY) played a role in plant resistance to stress. However, little is known about the GLY in pear.

**Results:**

Here, a total of 57 *PbrGLY* genes were identified through homologous comparison and analysis of conserved structural domains, which are unevenly distributed across pear chromosomes. Phylogenetic analysis revealed that the PbrGLY family can be divided into three main subfamilies, with varying numbers of members in each. Gene and protein structure analysis showed that PbrGLY possess a different number of exons and conserved motifs, and their promoter regions contain multiple stress-responsive and hormone-responsive elements. qRT-PCR analysis found that the expression levels of *PbrGLY* significantly changed after in response to *B. dothidea* infection. The transient silencing of the *PbrGLYI-28* gene increased the susceptibility and methylglyoxal content of pear to *B. dothidea*, and decreased GLY activity of pear. The content of H_2_O_2_ and O_2_^−^ was higher in TRV2-*PbrGLYI-28* leaves than that in TRV2 leaves. The antioxidant enzyme activity and pathogen resistance related gene expression was lower in TRV2-*PbrGLYI-28* leaves than that in TRV2 leaves.

**Conclusion:**

This study speculates that the *PbrGLY* family may functionally differentiate and coordinately regulate pear resistance to ring rot disease, with the expression changes of *PbrGLYI-28* potentially associated with *B. dothidea* infection and pear resistance.

**Supplementary Information:**

The online version contains supplementary material available at 10.1186/s12870-025-06302-6.

## Background

China is one of the centers of origin for the *Pyrus* genus, with a cultivation history of over 3000 years, making it the third largest fruit crop after apples and citrus. Its cultivation area, production, and export volumes all rank first in the world, holding a dominant position in the global pear industry. Pear ring rot is a fungal disease caused by *Botryosphaeria dothidea* (*B. dothidea*), which primarily harms the trunk, fruit, and leaves of pear trees, leading to rough bark, premature tree decline, and severely affecting the yield and quality of pear fruit [1]. However, the mechanism by which pear resist *B. dothidea* is not yet clear.

The glyoxalase system is the main pathway for clearing methylglyoxal (MG), converting it into lactic acid, and participating in the regulation of plant growth, development, and stress resistance. The traditional glyoxalase system consists of glyoxalase I (GLYI; lactoylglutathione lyase) and glyoxalase II (GLYII; hydroxyacylglutathione hydrolase), which rely on glutathione to clear MG [2]. GLYIII is a novel glyoxalase that operates independently of the traditional glyoxalase system, capable of directly catalyzing the irreversible conversion of MG into lactic acid without glutathione or other cofactors [3, 4]. Currently, the glyoxalase family has been identified in various model crops, including *Arabidopsis*, rice, soybean, and grape [3, 5, 6]. These species’ *GLY* genes have multiple subcellular localization members. *GLYI* is divided into Ni^2+^-dependent and Zn^2+^-dependent types, while GLYII belongs to the β-lactamase protein family, featuring a binuclear metal center composed of Fe^3+^, Zn^2+^, and Mn^2+^. GLYIII belongs to the DJ-1/PfpI superfamily, achieving optimal activity without metal ions [3, 7, 8]. During plant growth and development, abiotic stresses and　biotic stresses such as bacterial, fungal, viral, and insect invasions are inevitable, and glyoxalase genes show high sensitivity to these stresses. Studies have found that after rice is infected with blast fungus, the expression level of *GLYI* genes decreases [9]. In wheat, the expression of *GLYI* genes is induced by *Fusarium graminearum*, peaking 12 h after inoculation, indicating its significant role in disease development [10]. Additionally, compared to wild-type rice, transgenic rice overexpressing cecropin A (a resistance gene against blast fungus) significantly increased the expression level of *GLYI* genes by about six times [11]. Currently, research on the role of glyoxalase genes in the response to biotic stress has primarily focused on GLYI, while studies on GLYII and GLYIII have mainly concentrated on abiotic stress responses. The recent discovery of the GLYIII enzyme, which serves as an alternative to the traditional glyoxalase system, has established a more efficient pathway for MG detoxification [12]. The upregulation of GLYII genes in tobacco and rice markedly improves tolerance to elevated levels of MG and NaCl [13]. The glutathione-independent tomato glyoxalase III2 (*SlGLYIII2*), significantly enhances salt and osmotic stress tolerance [14]. Transgenic sugarcane overexpressing the *GLYIII* gene from sugarcane exhibits enhanced tolerance to salinity stress [15].

As a globally cultivated crop, the economic value of pears is significant; however, research on *GLYI*, *GLYII*, and *GLYIII* genes has not been fully explored. Therefore, the objective of this study is to systematically identify and analyze these genes to accelerate the progress of research on pear resistance to ring rot disease. This study aims to identify the *PbrGLY* gene family in pears at the whole-genome level, including chromosome localization, phylogenetic tree construction, collinearity analysis, conserved domain analysis, and cis-acting element analysis. Additionally, this study applied qPCR technology to investigate the expression patterns of these gene members under *B. dothidea* infection. This comprehensive analysis of the glyoxalase gene family in pears aims to identify key genes resistant to *B. dothidea* and provide new strategies for the prevention and control of *B. dothidea* in pear trees, thereby providing a scientific basis for improving the yield and quality of pear fruit, which is of great significance for promoting the sustainable development of the pear industry.

## Methods and materials

### Plant materials and treatments

For inoculating with *B. dothidea*, leaves of the *Pyrus bretschneideri* ‘Dangshan Suli’ cultivar grown in Hushu orchard of Nanjing Agricultural University, Nanjing, China, were initially disinfected with a 0.1% sodium hypochlorite solution for a duration of 10 min, followed by triple rinsing with sterile water. Subsequently, each leaf was pricked at four locations using a sterile needle. The inoculation process involved the application of mycelial agar pieces (5 mm in diameter), obtained from the peripheral regions of vigorous *B. dothidea* colonies cultivated on potato dextrose agar (PDA, Solarbio, Beijing, China). Leaf tissues excluding the diseased spots were sampled at intervals of 0, 2, 4, 6, and 8 days post-inoculation (dpi), with each set comprising five leaves and three such sets, summing up to 15 leaves for each time point. The leaf specimens were rapidly frozen in liquid nitrogen and preserved at -80 °C for future analysis [16].

### Screening and phylogenetic tree construction of the *PbrGLY* gene family

The HMM models of the conserved domains of the GLYI family were searched and downloaded (lactoylglutathione lyase domain, PF00903), the GLYII family (metallo-betalactamase domain, PF00753), and the GLYIII family (DJ-1/PfpI, PF01965) from the Pfam database (http://pfam.xfam.org/). Subsequently, we utilized these domain models to search for candidate *GLY* genes in the *Pyrus bretschneideri* ‘Dangshan Suli’ genome database (http://peargenome.njau.edu.cn) using HMMER search. Finally, we performed homologous alignment, removed redundant genes, and retained purified *PbrGLY* sequences.

A total of 57 PbrGLY protein sequences, along with GLY protein sequences from *Arabidopsis* (https://www.arabidopsis.org/) [17], *Oryza sativa* (https://rice.uga.edu/downloads_gad.shtml) [17] and *Vitis vinifera* (https://www.uniprot.org/) [5]. We employed ClustalW to conduct a multiple sequence alignment of the GLY protein sequences from four plant species. Subsequently, phylogenetic analysis was performed utilizing MEGA 11, with the neighbor-joining (NJ) method selected to construct the GLY phylogenetic tree. The specific parameters were as follows: the p-distance parameter was set, and bootstrap replicates were conducted 1000 times. The phylogenetic tree was then beautified using the online tool EVOLVIEW (https://www.evolgenius.info/evolview/#/).

### Physicochemical properties, structural, and conserved motif analysis of PbrGLY

By leveraging the annotated genome files of the *Pyrus bretschneideri* ‘Dangshan Suli’, we analyzed the lengths of cDNA and CDS for each member of the *PbrGLY* gene family. The Protein Parameter Calc feature within TBtools was utilized to assess various attributes of the encoded proteins, including their length, molecular weight, instability index, theoretical isoelectric point, and overall average hydropathy [18]. Information on exons and introns of the *PbrGLY* genes was extracted from the *Pyrus bretschneideri* genome annotation files, and the Visualiza Gene Structure functionality in TBtools was used to create diagrams illustrating the gene structures. Motif analysis of the PbrGLY protein in pear was carried out using MEME (https://meme-suite.org/meme/tools/meme). The specific parameters were set as follows: the motif length was defined as 3–26 residues, with a significance threshold e-value < 1e-5, and the expected number of motifs was set to 10. The Gene Structure View function in TBtools was employed to visualize the distribution of the motifs.

### Chromosome localization, collinearity, and cis-acting element analysis of *PbrGLY*

To visualize the chromosomal positions and tandem repeats of PbrGLY, we utilized the BLAST and MCScanX functions in TBtools with pear protein sequences and PbrGLY protein sequences. We obtained files for chromosome length and gene density using the Fasta Stats and Fasta stats table row features in TBtools, based on the pear genome file and annotation file. The positions of the *PbrGLY* gene family on the chromosomes were then extracted using the Text Block Extract and Filter function. Collinearity information files within the pear species for PbrGLY were acquired through the One Step MCScanX-Super Fast and Gene Position Extract functions, and the collinearity of PbrGLY was visualized using the chromosome length file, target gene position file, and collinearity information file in Advanced Circos.

The upstream promoter sequences of the *PbrGLY* gene family in the pear genome were extracted using TBtools software. The online software PlantCARE was employed to predict cis-acting elements, and the types and quantities of these elements for each gene were calculated.

### qRT-PCR analysis of *PbrGLY* expression levels

Total RNA was extracted using a plant RNA extraction kit [19]. Total RNA was reverse transcribed using the PrimeScript reverse transcription kit (TaKaRa, Dalian, China). After reverse transcription of total RNA to cDNA, qRT-PCR was performed using the StepOnePlus™ RT-PCR system and SYBR Premix Ex Taq™ (TaKaRa, Dalian, China) on the LightCycler 480 (Roche, USA). Tubulin was used as the reference gene, as shown in Table. S1. The 2^−ΔΔCT^ method was used to calculate the relative expression levels of the genes [20].

### Vector construction and plant transformation

Utilizing virus-induced gene silencing (VIGS), we employed tobacco rattle virus (TRV)-based vector constructs (pTRV1/2) as described [16]. In the development of the pTRV-*PbrGLYI-28* vector, a PCR amplification was conducted to obtain a segment of the *PbrGLYI-28* open reading frame (ORF), subsequently integrated into the pTRV2 vector. The *Agrobacterium* strain containing the constructed pTRV2/pTRV2-PbrGLYI-28 was cultured in large quantities until it reached an optical density (OD600) of 0.6–0.8, after which the bacterial cells were collected. The cells were resuspended in infiltration buffer (10 mM MgCl_2_, 10 mM MES (pH 5.5), and 150 µM acetosyringone) and induced at room temperature (25℃) for 4 h. The pTRV2-*PbrGLYI-28* and pTRV2 empty vector were mixed with pTRV1 buffer in a 1:1 volume ratio and injected into the leaves of approximately 45-day-old *Pyrus betulaefolia* seedlings grown in Hushu orchard of Nanjing Agricultural University, Nanjing, China, using a micro-injector, typically selecting the lower leaves to facilitate the spread of the viral vector. The seedlings were kept in the dark for 24–36 h, followed by normal growth conditions (25℃, 16:8 h light: dark) for 1 week, after which the silencing efficiency and expression levels of the target genes were assessed. Seedlings with silenced target genes were selected, and their leaves were cut and inoculated with *B.dothidea*. The diameter of the disease spots was measured and photographed 4 days post-inoculation.The entire leaf tissue excluding the diseased spots was sampled at 4 days post-inoculation. The specific primers used are detailed in Supplementary Table S1.

### Physiological measurement

ROS levels (H_2_O_2_ and O_2_^−^) and antioxidant enzyme activities were measured using specific assay kits (Comin, Suzhou, China).

### Statistical analysis

Results are mean ± SE from three replicates and analyzed by ANOVA with Tukey’s test (*P* < 0.05) using SPSS18 (IBM SPSS Statistics, Chicago, IL, USA).

## Results

### Physicochemical property analysis of PbrGLY genes

A total of 57 PbrGLY family members were identified in the genome of the *Pyrus bretschneideri*. In pear, the *PbrGLY* family encompasses three gene subfamilies, namely *PbrGLYI*, *PbrGLYII*, and *PbrGLYIII*. As shown in Table 1, the analysis of the physicochemical characteristics of PbrGLY proteins revealed that the longest protein consists of 1251 amino acids (AA) (PbrGLYI-10), and the shortest consists of 84 AA (PbrGLYI-26). The molecular weight range is from 9198.49 Da (PbrGLYI-26) to 139290.17 Da (PbrGLYI-10), and the isoelectric point range is from 4.65 (PbrGLYI-17) to 9.6 (PbrGLYI-26). Additionally, among the PbrGLYI subfamily, except for PbrGLYI-12, -16, -21, and − 24 which are hydrophobic proteins, the rest are hydrophilic; all PbrGLYII proteins are hydrophilic; and all PbrGLYIII proteins are hydrophobic.

**Table 1:** Molecular characteristics of PbrGLY gene family proteins

Gene name	Gene ID	Protein size(aa)	MW(Da)	pI	Instability Index	Aliphatic Index	GRAVY	Localization prediction
PbrGLYI-1	Pbr039832.1	236	26235.77	6.9	37.18	66.57	-0.36	Chloroplast
PbrGLYI-2	Pbr042834.1	124	13921.56	5.14	23.08	60.56	-0.544	Cytoplasmic
PbrGLYI-3	Pbr026416.1	168	19410.23	9.08	50.11	63.75	-0.676	Nuclear
PbrGLYI-4	Pbr027554.1	362	39628.5	6.39	32.21	86.22	-0.168	Chloroplast
PbrGLYI-5	Pbr009212.1	362	39714.2	6.07	34.89	85.69	-0.316	Chloroplast
PbrGLYI-6	Pbr012590.2	141	15386.41	9.45	32.97	74.18	-0.704	Nuclear
PbrGLYI-7	Pbr015491.1	641	72069.48	8.73	36.24	80.17	-0.383	Mitochondrial
PbrGLYI-8	Pbr000137.1	292	32765.27	5.36	31.79	86.47	-0.392	Cytoplasmic
PbrGLYI-9	Pbr031905.1	292	32759.41	5.47	29.92	87.12	-0.364	Cytoplasmic
PbrGLYI-10	Pbr039725.2	1251	139290.17	9.18	54.58	72.45	-0.649	Nuclear
PbrGLYI-11	Pbr001024.1	172	19513.6	5.65	66.36	87.79	-0.208	Cytoplasmic
PbrGLYI-12	Pbr040186.1	206	22929.57	9.08	38.13	88.5	0.023	Plasma Membrane
PbrGLYI-13	Pbr003056.1	172	19569.66	5.47	68.6	88.37	-0.222	Cytoplasmic
PbrGLYI-14	Pbr010603.1	174	19759.66	7.65	67.29	78.45	-0.497	Nuclear
PbrGLYI-15	Pbr022133.1	184	20928.19	4.86	50.28	85.76	0.015	Extracellular
PbrGLYI-16	Pbr029420.1	184	20945.31	5.19	46.12	86.3	0.039	Extracellular
PbrGLYI-17	Pbr009688.1	214	24694.42	4.65	48.49	68.74	-0.942	Nuclear
PbrGLYI-18	Pbr026568.1	153	16802.13	5.07	45.27	80.85	-0.139	Nuclear
PbrGLYI-19	Pbr004428.1	197	21727.98	8.31	43.95	94.57	-0.31	Mitochondrial
PbrGLYI-20	Pbr041492.1	237	26589.77	8.24	43.32	97.89	-0.142	Mitochondrial
PbrGLYI-21	Pbr025826.1	140	15596.69	6.29	46.71	77.36	-0.29	Cytoplasmic
PbrGLYI-22	Pbr009307.1	117	12898.03	6.96	42.56	93.42	0.038	Mitochondrial
PbrGLYI-23	Pbr026735.1	120	13237.48	8.62	39.5	91.92	0.022	Mitochondrial
PbrGLYI-24	Pbr020837.1	143	15781.62	5.89	30.27	69.51	-0.428	Cytoplasmic
PbrGLYI-25	Pbr034015.1	84	9198.49	9.6	43.35	84.88	-0.117	Mitochondrial
PbrGLYI-26	Pbr010673.1	440	48539.51	5.56	35.85	74.7	-0.332	Cytoplasmic
PbrGLYI-27	Pbr002375.1	440	48595.62	5.56	36.01	75.36	-0.322	Cytoplasmic
PbrGLYI-28	Pbr024160.1	175	18815.45	4.94	29.23	88.17	-0.007	Cytoplasmic
PbrGLYII-1	Pbr001406.1	258	28750.64	6	35.89	83.1	-0.428	Cytoplasmic
PbrGLYII-2	Pbr002378.1	299	32799.66	7.69	34.42	86.09	-0.161	Mitochondrial
PbrGLYII-3	Pbr003191.1	669	74870.07	6.19	38.57	93.2	-0.127	Cytoplasmic
PbrGLYII-4	Pbr005205.2	351	39449.56	8.67	50.01	99.46	-0.217	Mitochondrial
PbrGLYII-5	Pbr007993.1	277	30289.5	6.59	32.19	86.97	-0.182	Mitochondrial
PbrGLYII-6	Pbr011475.1	298	33101	6.18	46.72	94.19	-0.133	Cytoplasmic
PbrGLYII-7	Pbr012123.1	1028	114986.16	8.84	38.57	75.32	-0.49	Nuclear
PbrGLYII-8	Pbr013325.1	427	47015.49	5.25	43.51	89.02	-0.147	Cytoplasmic
PbrGLYII-9	Pbr017223.1	186	20783.69	6.28	50.21	81.77	-0.218	Cytoplasmic
PbrGLYII-10	Pbr017741.1	933	102971.51	7.86	43.19	84.13	-0.37	Nuclear
PbrGLYII-11	Pbr041014.1	933	102898.43	7.6	43.94	84.54	-0.367	Nuclear
PbrGLYII-12	Pbr019935.2	737	82207.67	6.01	45.85	73.15	-0.487	Nuclear
PbrGLYII-13	Pbr026543.1	696	77701.15	6.26	41.95	90.85	-0.199	Cytoplasmic
PbrGLYII-14	Pbr028803.1	535	59699.13	6.37	42.46	87.1	-0.188	Cytoplasmic
PbrGLYII-15	Pbr029630.1	296	32521.66	8.95	34.54	91.55	-0.103	Mitochondrial
PbrGLYII-16	Pbr031338.1	711	79078.36	4.92	32.77	86.77	-0.249	Cytoplasmic
PbrGLYII-17	Pbr032495.2	362	40545.5	6.79	49.3	89.94	-0.148	Chloroplast
PbrGLYII-18	Pbr032813.1	329	36180.3	7.65	45.95	81.25	-0.257	Mitochondrial
PbrGLYII-19	Pbr035360.1	740	82317.29	5.03	35.46	88.77	-0.229	Cytoplasmic
PbrGLYII-20	Pbr036910.1	893	98673.43	7.88	43.8	84.5	-0.418	Nuclear
PbrGLYII-21	Pbr039621.1	314	35068.29	5.78	39.38	99.04	-0.052	Cytoplasmic
PbrGLYIII-1	Pbr025832.1	396	41980.75	5.63	31.76	108.13	0.174	Cytoplasmic
PbrGLYIII-2	Pbr029022.1	249	26787.76	5.44	48.02	98.67	0.062	Cytoplasmic
PbrGLYIII-3	Pbr033581.1	392	41842.08	4.95	37.13	90.15	0.133	Cytoplasmic
PbrGLYIII-4	Pbr033582.1	389	41849.09	5.22	39.57	94.81	0.17	Cytoplasmic
PbrGLYIII-5	Pbr034371.1	396	42050.92	5.79	31.76	109.12	0.179	Cytoplasmic
PbrGLYIII-6	Pbr038224.1	435	46390.09	8.04	37.17	101.13	0.091	Chloroplast
PbrGLYIII-7	Pbr040391.1	317	34184.54	8.66	43.7	106.44	0.053	Mitochondrial
PbrGLYIII-8	Pbr040392.1	123	12685.72	9.22	36.67	83.33	0.254	Chloroplast

### Phylogenetic tree of the *GLY*

**Fig. 1:**
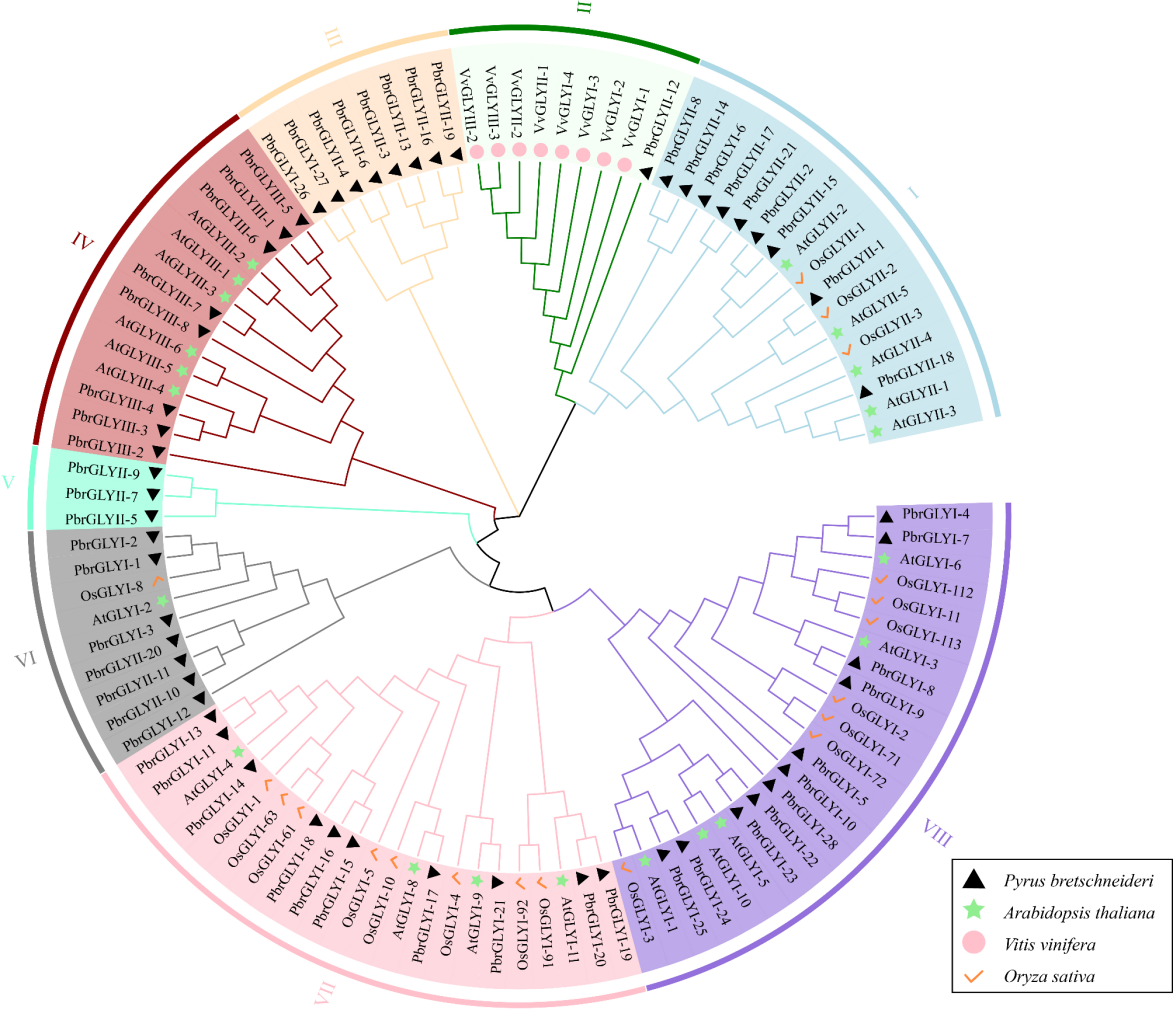
Phylogenetic and evolutionary analysis of GLY proteins from *Pyrus bretschneideri*,* Arabidopsis*, rice and grape. The phylogenetic tree was constructed using GLY protein sequences and the neighbor-joining algorithm method

To gain a deeper understanding of the evolutionary relationships and classification of plant GLY proteins, this study collected a total of 107 GLY protein sequences from *Arabidopsis*, *Oryza sativa*, *Pyrus bretschneideri*, and *Vitis vinifera*, and constructed a phylogenetic tree. As shown in Fig. 1, based on the topology of the phylogenetic tree, nodes forming distinct independent branches were classified into subgroups, identifying a total of eight GLY protein subgroups. In the constructed phylogenetic tree, Subgroup VIII is the largest, containing more than 23 protein sequences; in contrast, Subgroup V is the smallest, containing only three protein sequences. Among them, most PbrGLYII proteins clustered with AtGLYII proteins on the same branch (Subgroup I), which also included some OsGLYII proteins. This indicates that PbrGLYII proteins have a closer phylogenetic relationship with *Arabidopsis* and *Oryza sativa* GLYII proteins; In Subgroup II, all VvGLY proteins and PbrGLYII-12 proteins were classified into the same branch, indicating a closer phylogenetic relationship between PbrGLYII-12 and *Vitis vinifera* GLY proteins; Subgroups III and V have similar distributions, both containing only PbrGLY proteins; In Subgroups IV, VI, VII, and VIII, analysis of secondary branches showed that most PbrGLY and AtGLY family members did not appear as orthologous gene pairs but instead exhibited the phenomenon of multiple paralogous genes clustering within the same species. This finding suggests that the evolution of *Pyrus bretschneideri* and *Arabidopsis* GLY family members within these subgroups is not entirely synchronous.

### Chromosome localization and collinearity analysis of *PbrGLY*

**Fig. 2:**
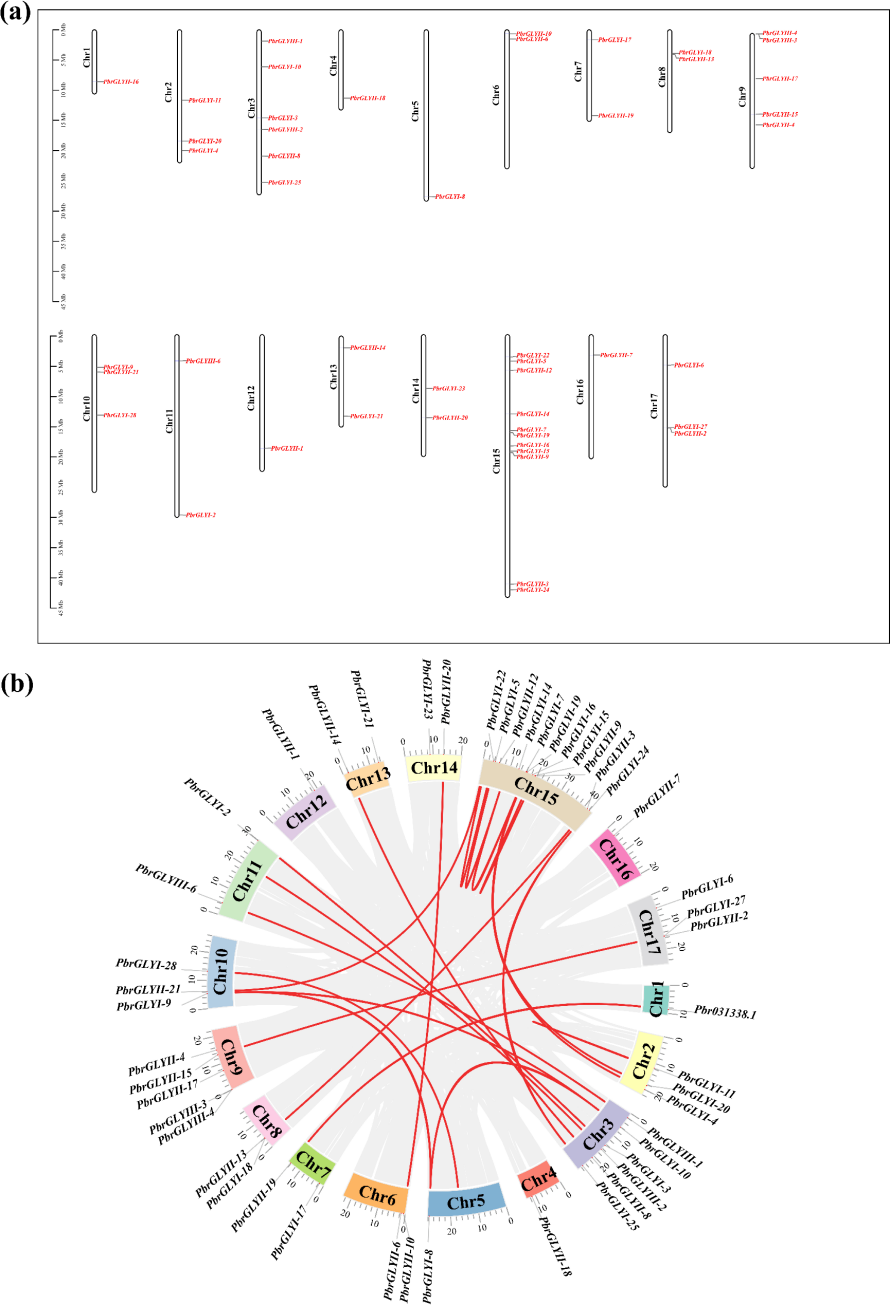
The chromosomal mapping and synteny analysis of *PbrGLY* genes across pear chromosomes. (**a**) The chromosomal location of *PbrGLY*. The chromosome number is indicated at the left of each chromosome and the scale is shown on the left. (**b**) Synteny analysis of *PbrGLY.* The genomes of *Pyrus bretschneideri* exhibit colinear blocks, as indicated by the gray lines, with the red line specifically delineating the colinear *PbrGLY* gene pairs

An analysis of the chromosomal (Chromosome, Chr) positions of the 57 *PbrGLY* family members in the *Pyrus bretschneideri* genome was conducted. The results revealed that, excluding the 9 *PbrGLY* that were not labeled on the chromosomes, the remaining 46 members were irregularly distributed across the pear chromosomes (Fig. 2a). The *PbrGLY* are predominantly concentrated on chromosomes Chr3, Chr5, and Chr9. Notably, chromosome Chr5 harbors the highest number of *PbrGLY* genes, including 11 *PbrGLY* genes (*PbrGLYI-*5, *PbrGLYI-7*, *PbrGLYI-14*, *PbrGLYI-15*, *PbrGLYI-16*, *PbrGLYI-19*, *PbrGLYI-2*2, *PbrGLYI-24*, *PbrGLYII-3*, *PbrGLYII-9*, and *PbrGLYII-12*). Collinearity analysis indicated that there are 28 pairs of *PbrGLY* genes that are collinear (Fig. 2b).

### Gene structure and protein motif analysis of the *PbrGLY* family

**Fig. 3:**
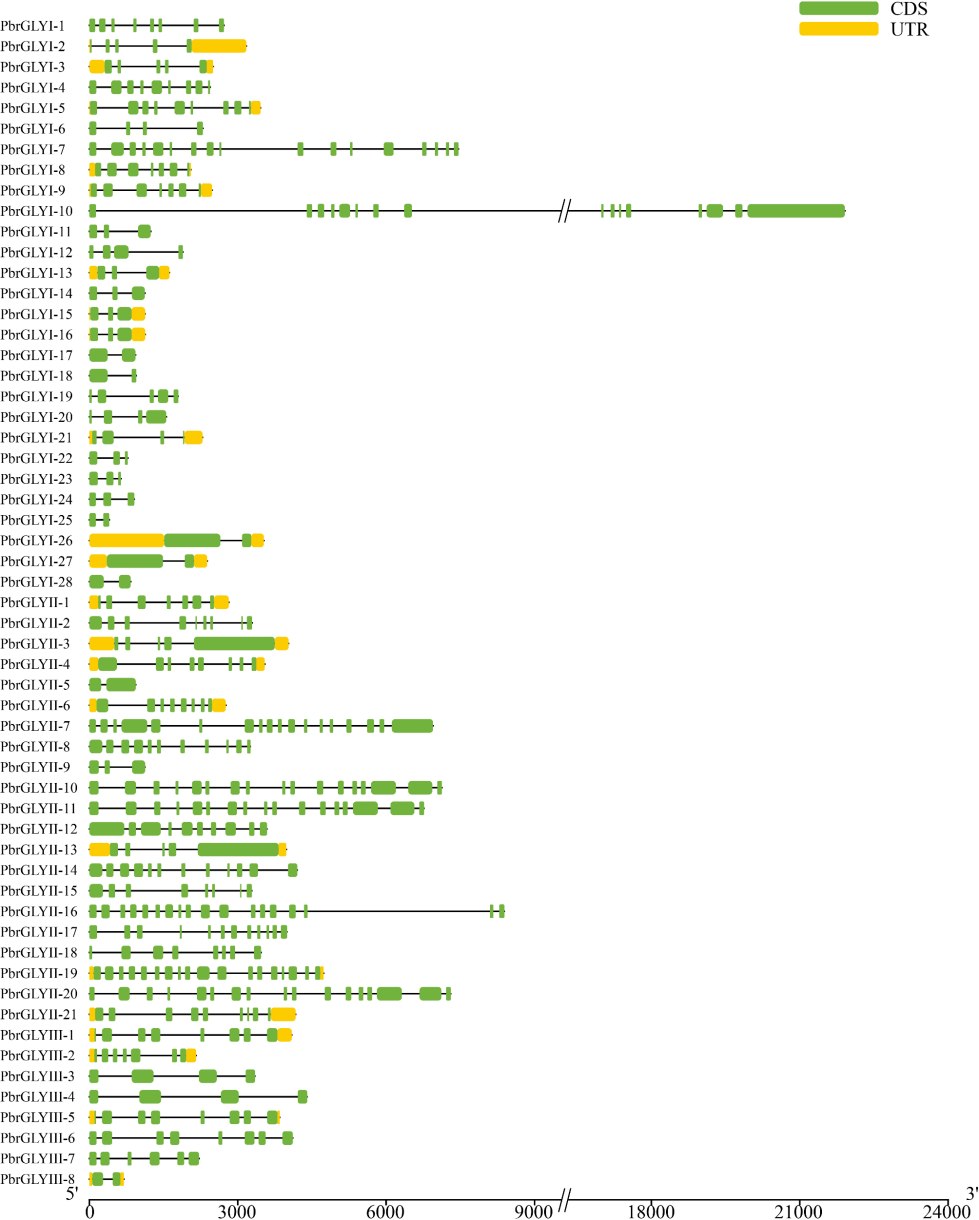
Gene structure of the *PbrGLY* in pear. The green boxes, dark lines, and red boxes represent exons, introns, and upstream/downstream sequences, respectively

The *PbrGLY* exhibits significant variation in exon/intron structure. Notably, *PbrGLYII-19* has the highest number of exons (18), while *PbrGLYI-17*, *PbrGLYI-18*, *PbrGLYI-25*, *PbrGLYII-5*, and *PbrGLYIII-8* have only 2 exons each (Fig. 3). Longer exons tend to encode larger proteins, potentially containing more functional domains, which may confer an advantage in the process of natural selection. Additionally, homologous genes have similar exon/intron structures; for example, *PbrGLYII-10/-11* and *PbrGLYIII-5/-6* both have the same number of exons and introns and share similar gene structures. Although there are clear differences in gene length and the number of introns/exons among *PbrGLY* family members, the gene structures within each subfamily are relatively similar. The similar gene structures and motifs within each subfamily further validate the classification credibility.

**Fig. 4:**
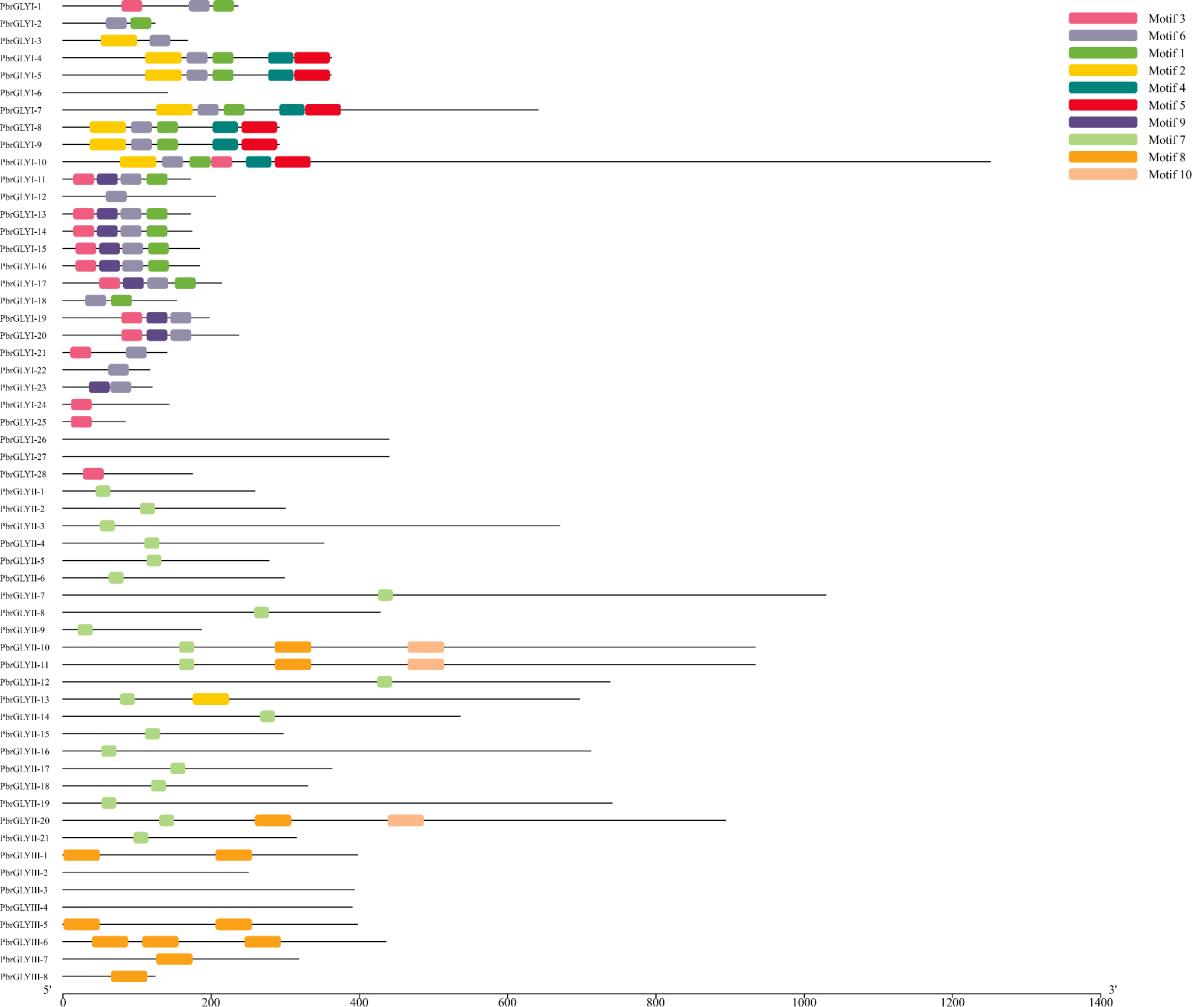
Schematic representation of domain architecture of *PbrGLY* genes in pear

PbrGLY family proteins contain 1–10 motifs (Fig. 4), with certain motifs being specific to different subfamilies. For instance, motifs 1–6 and 9 are exclusive to PbrGLYI, motif 7 is only present in PbrGLYII, and motifs 8 are found only in PbrGLYII-10, -11, and PbrGLYIII. The presence of these specific motifs in different subfamilies indicates their unique roles within each subfamily. Within the same subfamily, the arrangement and number of motifs are highly conserved. For example, most PbrGLYI members contain motifs 1, 3, 6, and 9, with a consistent order, and all PbrGLYII members possess motif 7. This specificity of subfamily motifs reflects the conservation in gene evolution.

### Analysis of cis-acting elements in *PbrGLY*

**Fig. 5:**
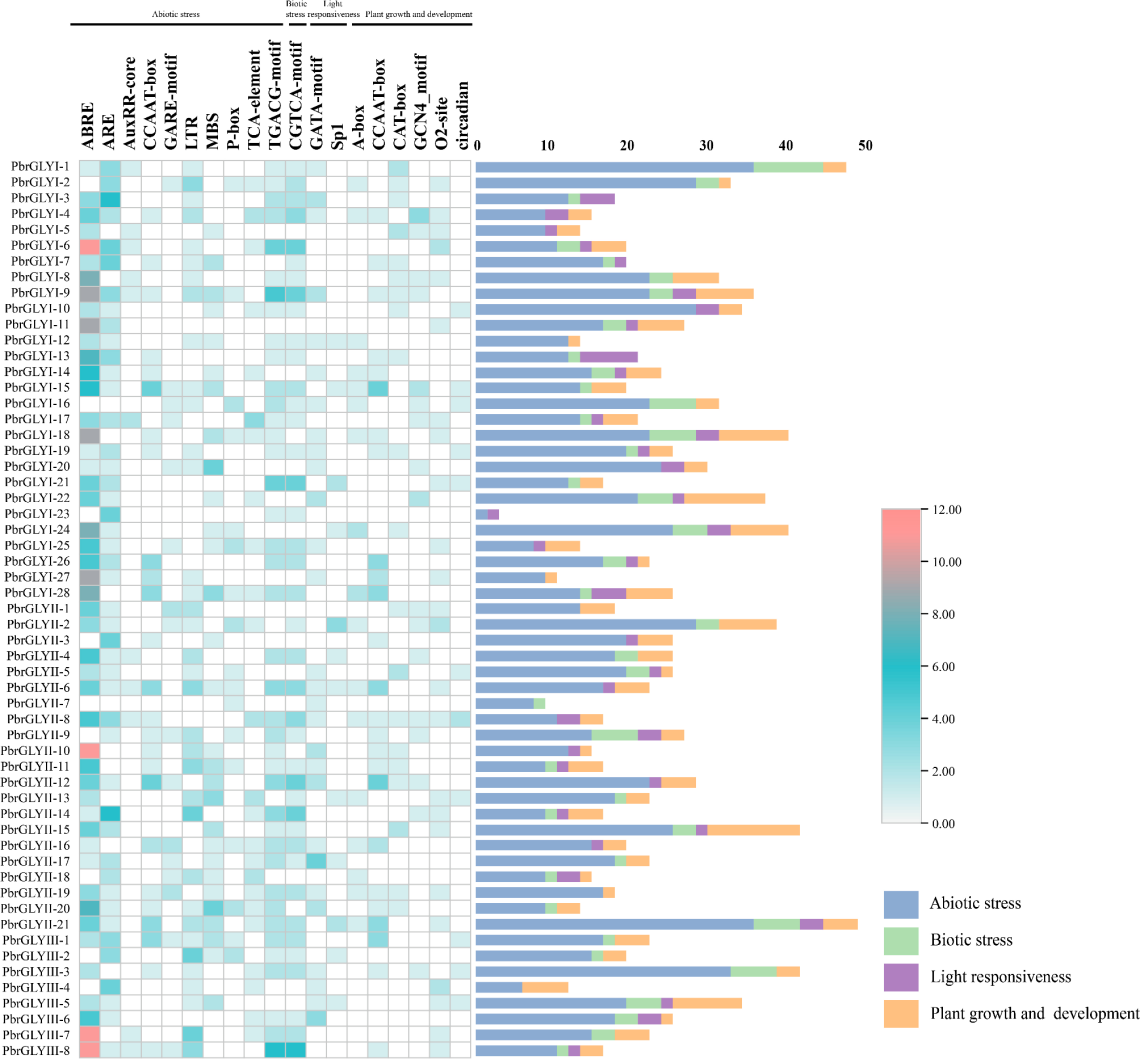
Analysis of cis-acting elements in the *PbrGLY.* The number of different cis-acting elements of *PbrGLY* is represented by a heat map

The promoter region of the *PbrGLY* family members, specifically the − 2000 bp sequence upstream of the non-coding region, was analyzed for cis-acting elements (Fig. 5). The results indicated that there are over 50 cis-acting elements within the *PbrGLY* family, which can be categorized into four major types based on their known functions: abiotic stress, biotic stress, light response, and plant growth and development. Analysis of the promoter regions of the *PbrGLY* genes revealed several stress-responsive cis-acting elements, such as the abscisic acid-responsive element (ABRE), anaerobic response element (ARE), salicylic acid-responsive element (TCA element), and methyl jasmonate-responsive element (TGACG motif).

### Expression analysis of *PbrGLY*

**Fig. 6:**
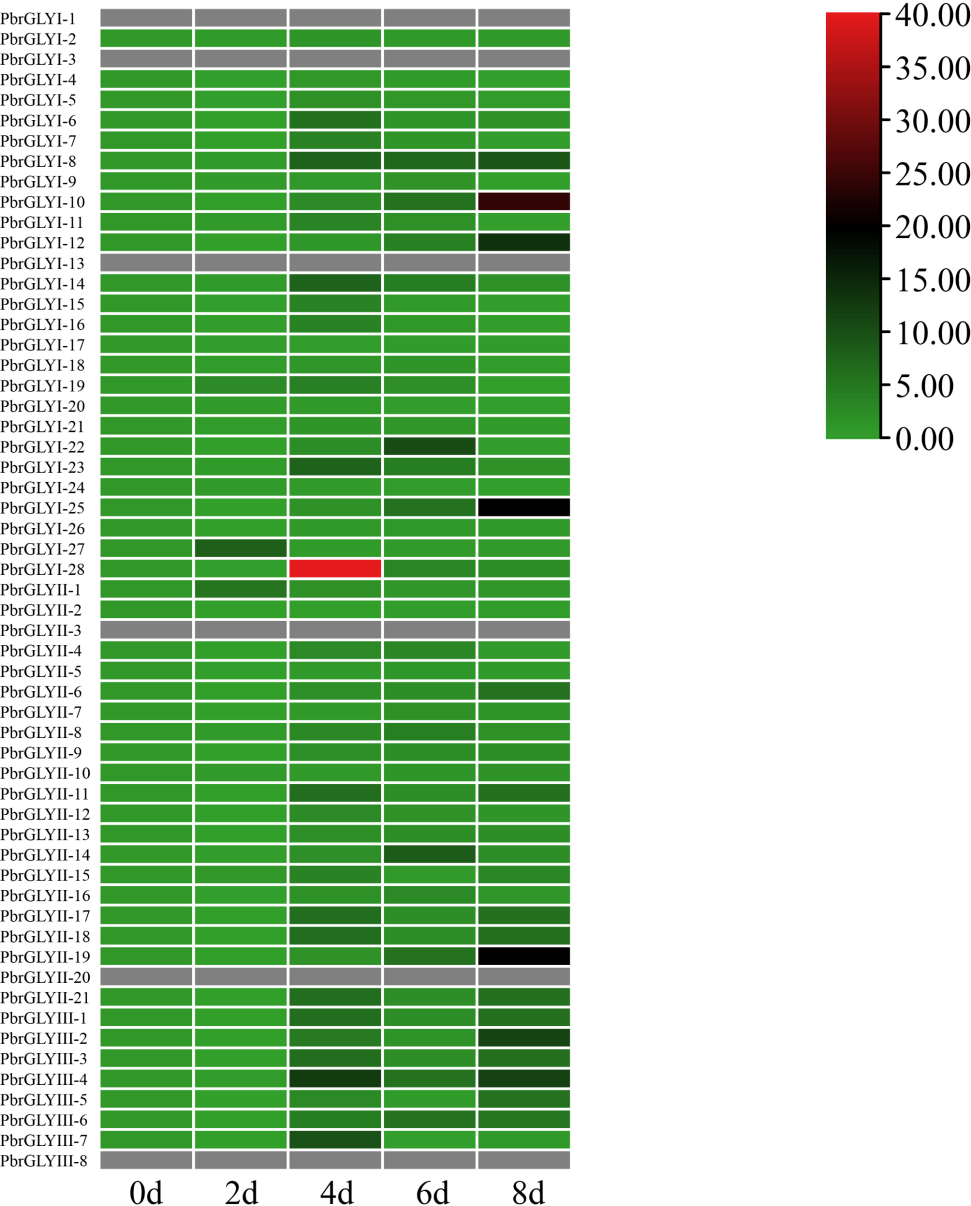
Expression of *PbrGLY* in response to *B. dothidea* infection. Expression levels were calculated relative to the expression of *Tubulin*. Expression of *PbrGLY* at 0 d was set to “1”

To elucidate the role of *PbrGLY* in the infection process of *B. dothidea*, we analyzed the expression of *PbrGLY* in DangshanSuli (*Pyrus bretschneideri*) leaves after infection with *B. dothidea*. To compare the expression specificity of the three subfamily members, we conducted a visual analysis of the *PbrGLY* under different time treatments of *B. dothidea*. As shown in Fig. 6, members of the pear *PbrGLY* gene family exhibited variations in expression levels at different time points during infection by *B. dothidea* (0, 2, 4, 6and 8 days). Specifically, grey indicates no expression, green represents low expression levels, black denotes moderate expression levels, and red signifies high expression levels. Within the *PbrGLYI* subfamily, *PbrGLYI-8*, *PbrGLYI-10*, *PbrGLYI-12* and *PbrGLYI-25* all exhibited high expression levels at 8 days post-infection (dpi). The overall trend suggests that expression levels increased with prolonged infection by *B. dothidea*. Notably, *PbrGLYI-28* displayed significantly higher expression levels at 4 dpi compared to other genes. In the *PbrGLYII* subfamily, *PbrGLYII-19* showed high expression levels at 8 dpi, while other genes such as *PbrGLYII-11*, *PbrGLYII-17* and *PbrGLYII-18* exhibited moderately high expression levels at 4 and 8 dpi, with similar values. Within the *PbrGLYIII* subfamily, *PbrGLYIII-4* demonstrated high expression levels from 4 to 8 dpi, and other genes like *PbrGLYIII-1*, *PbrGLYIII-*2 and *PbrGLYIII-8* exhibited moderately high expression levels at 4 and 8 dpi, similar to the *PbrGLYII* subfamily. In summary, all *PbrGLY* genes were induced by *B. dothidea* and exhibited elevated expression levels, with most genes showing higher expression at 4 and 8 dpi. Notably, the expression level of *PbrGLYI-28* at 4 dpi was higher than that of any other gene at any treatment time, suggesting that *PbrGLYI-28* may play a crucial role in the resistance of pear to *B. dothidea* infection. Moreover, the diverse expression trends of the *PbrGLY* family during the response to *B. dothidea* infection indicate that they may fulfill different roles in this process.

### Transient silencing of *PbrGLYI-28* enhances pear susceptibility to *B. dothidea*

**Fig. 7:**
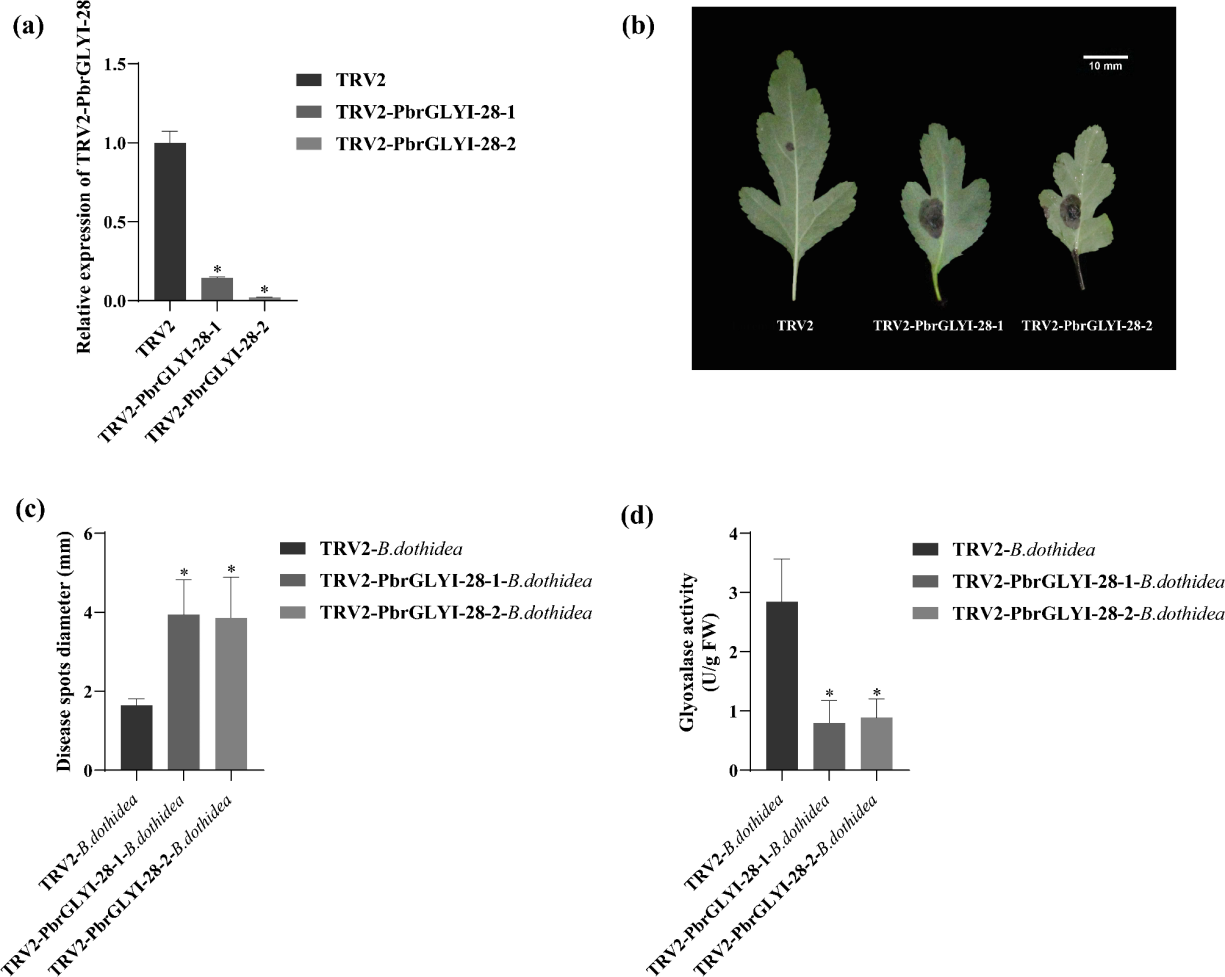
Assessment of resistance in *PbrGLYI-28*-silenced *Pyrus betulaefolia* to *B. dothidea* infection. (**a**) The expression level of *PbrGLYI-28* in *Pyrus betulaefolia*. (**b**) Phenotype of *PbrGLYI-28*-silenced plants after inoculation with *B. dothidea* for 4 days. Bars = 0.5 cm. (**c**) Diameter of disease spots of *PbrGLYI-28*-silenced plants. (**d**) Activity of glyoxalase in *PbrGLYI-28*-silenced plants. Data are means of three replicates with SE. Asterisks denote statistically significant differences between TRV2 and TRV2- *PbrGLYI-28* plants (*P* < 0.05, ANOVA)

In this study, we employed the VIGS (Virus-Induced Gene Silencing) technology to transiently silence *PbrGLYI-28* in *Pyrus betulaefolia*. The results indicated that the expression levels of *PbrGLYI-28* in the silenced plants (TRV2*-PbrGLYI-28*) were significantly lower than those in the control (TRV2) (Fig. 7a). Moreover, compared to TRV2, the TRV2-*PbrGLYI-28* plants exhibited a larger lesion diameter (Fig. 7b and c), suggesting that the resistance of pear plants to *B. dothidea* was compromised, and their susceptibility was enhanced when *PbrGLYI-28* was silenced. In addition, the glyoxalase activity of TRV2-*PbrGLYI-28* plants was obviously lower than that in TRV2 plants (Fig. 7d).

### Transient Silencing of *PbrGLYI-28* decreased antioxidase activity and pathogen-relative genes expression in Pear

Due to the changes of glyoxalase in the TRV2-*PbrGLYI-28* pear plants, we examined the MG content in the TRV2-*PbrGLYI-28* pear plants.

**Fig. 8:**
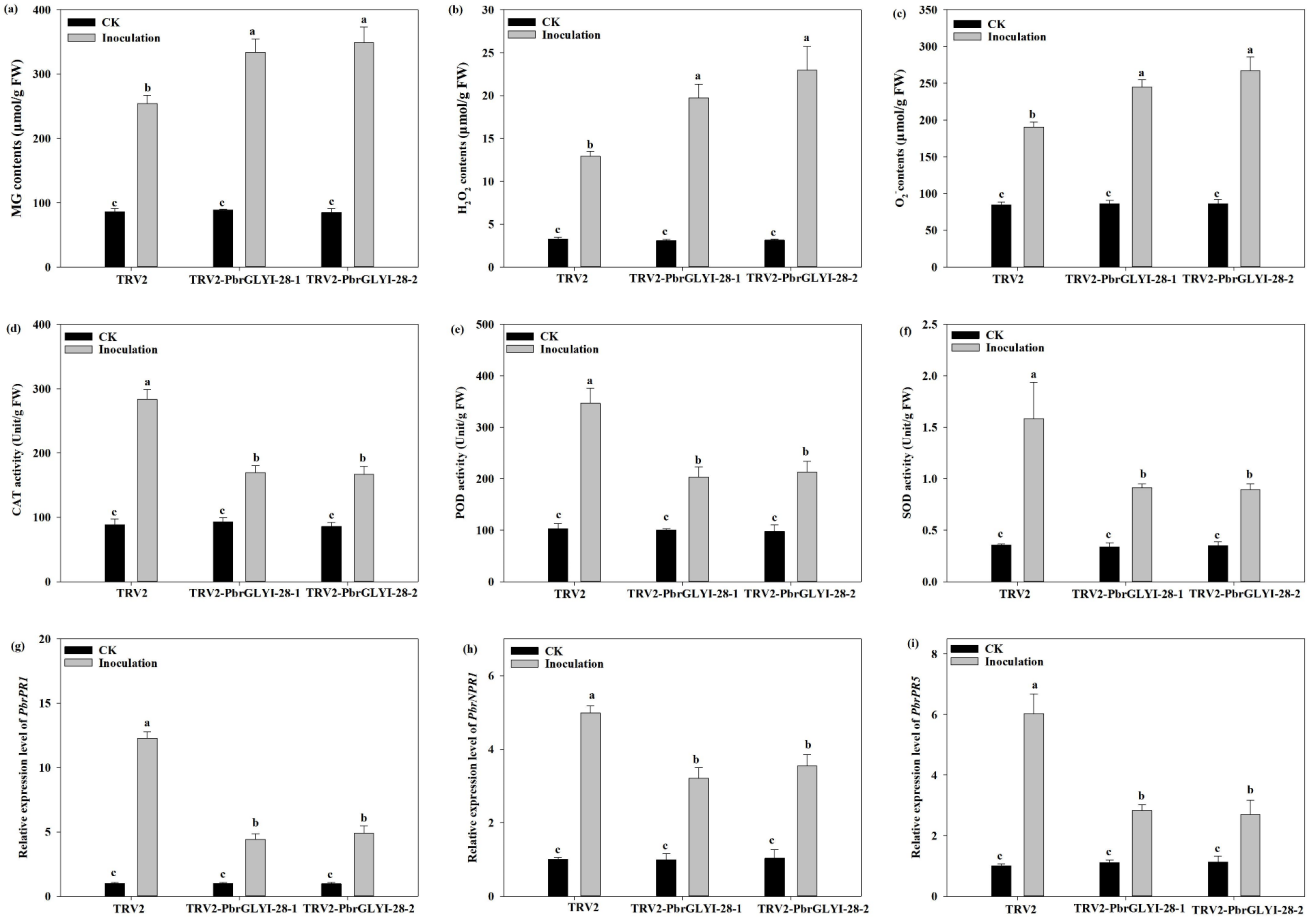
Changes in antioxidant enzyme activity and pathogen-related gene expression in *PbrGLYI-28*-silenced *Pyrus betulaefolia* under *B. dothidea* infection. The antioxidant enzyme activity and pathogen-related gene expression was accessed at 4 dpi. (**a**) MG content; (**b** and **c**) H_2_O_2_ and O_2_^−^ content; (**d**-**f**) CAT, POD and SOD activity; (**g**-**i**) Expression level of *PbrPR1*, *PbrNPR1* and *PbrPR5*. Expression of *PbrPR1*, *PbrNPR1* and *PbrPR* in TRV2 leaves under no-inoculation was set to “1”. Data are means of three replicates with SE. Different letter denote statistically significant differences between TRV2 and TRV2-*PbrGLYI-28* plants (*P* < 0.05, ANOVA)

A significant elevation in MG concentration was observed in TRV2-*PbrGLYI-28* pear plants compared to the control TRV2 plants when infected with *B. dothidea*, as depicted in Fig. 8a. Furthermore, the TRV2-*PbrGLYI-28* pear plants exhibited a pronounced accumulation of key reactive oxygen species (ROS) components, namely hydrogen peroxide (H_2_O_2_) and superoxide anion (O_2_^−^), following inoculation (Fig. 8b and c). The temporary silencing of *PbrGLYI-28* resulted in diminished antioxidant enzyme activities, specifically catalase (CAT), peroxidase (POD), and superoxide dismutase (SOD), during infection (Fig. 8d-f). Conversely, pathogen-associated genes, including *PbrPR1*, *PbrNPR1*, and *PbrPR5*, displayed heightened expression levels in the TRV2 plants relative to the TRV2-*PbrGLYI-28* pear plants under infection conditions (Fig. 8g-i).

## Discussion

MG, a reactive byproduct of sugar metabolism, originates from the non-enzymatic conversion of dihydroxyacetone phosphate (DHAP), a compound engaged in diverse metabolic pathways such as glycolysis, gluconeogenesis, and the Calvin-Benson-Bassham cycle [21, 22]. The glyoxalase I/II (GLY I/II) system, which utilizes reduced glutathione (GSH) as a cofactor, catalyzes the degradation of MG into D-lactate [23, 24]. The traditional glyoxalase system mainly refers to the glyoxalase I (GLYI; lactoylglutathione lyase) and glyoxalase II (GLYII; hydroxyacylglutathione hydrolase) that together form a glutathione-dependent system, playing a primary role in the clearance of MG. Meanwhile, GLYIII (or DJ-1) can directly catalyze the irreversible conversion of MG to lactic acid. In plant cells, the dynamic balance of methylglyoxal is maintained through the synergistic action of these systems. Therefore, the glyoxalase system not only clears excess MG to maintain its homeostasis but also maintains the redox balance in cells by regulating the regeneration of glutathione (GSH), playing an important role in plant perception, response, and adaptation to environmental stresses. Research has reported that the glyoxalase family has been identified in rice, *Arabidopsis*, soybean, grape, and alfalfa [3, 6, 7]. However, glyoxalase family genes have not been studied in pear. In this study, we conducted a comprehensive analysis of the *PbrGLY* gene family in *Pyrus bretschneideri*, revealing insights into their physicochemical properties, evolutionary relationships, chromosomal localization, gene structure, protein motifs, cis-acting elements, and their roles in response to infection by *B. dothide*a. Our findings provide a foundation for understanding the functional diversity and evolutionary dynamics of the *PbrGLY* family in pear.

In this study, a total of 57 *PbrGLY* gene family members were identified in the pear genome, mainly divided into three subfamilies, with 28 PbrGLYI, 21 PbrGLYII, and 8 PbrGLYIII family members, indicating that the GLYI family, which constitutes a dual-enzyme pathway, is much larger than the GLYII family. The number of *GLY* genes identified in *Arabidopsis*, rice, soybean, *Sorghum bicolor*, and oat was lower than that in pear, which may be related to genome duplication events during evolution [3, 25, 26].A recent study reported a whole genome duplication (WGD) event within the Maloideae family and a genomic replication event dating back to half a billion years ago in Rosaceae, which resulted in a chromosome count increase from nine to seventeen [27–29]. Our collinearity analysis has revealed the presence of 28 pairs of *PbrGLY* genes that exhibit collinearity. Post-duplication, these 28 pairs of collinear *PbrGLY* genes are hypothesized to experience significant functional divergence, potentially contributing to the emergence of novel functions, a key aspect of evolutionary development. This divergence in gene function following duplication events is crucial for understanding the adaptive radiation and diversification observed in the Rosaceae family.

The analysis of physicochemical properties indicates that the number of amino acids, protein molecular weight, and isoelectric point (pI) of PbrGLY are similar to those of soybean GLY protein members [3]. Differences in protein size and hydrophilicity among different subfamilies of PbrGLY may be related to their different gene functions. Chromosome localization and collinearity are also considered. Furthermore, differences in exon/intron structure and motif distribution among *PbrGLY* gene family members may be related to their functional divergence in biology.

Cis-acting elements play a crucial role in regulating plant gene expression and are indispensable for plant responses to environmental changes and evolution [30]. Studies have shown that members of the GLY family can respond to a variety of biotic (bacterial and fungal infections) and abiotic stresses (drought, extreme temperatures, heavy metals, and plant hormones) [10, 11, 31, 32]. Promoter analysis has revealed a rich diversity of cis-acting elements in the promoter regions of *PbrGLY*, including hormone response elements, biotic and abiotic stress response elements, and growth and development-related elements. Among them, the types of cis-acting elements related to biotic and abiotic stresses are the most abundant, and the presence and number of these response elements may determine the transcriptional levels and stress response capabilities of *PbrGLY* under different adverse conditions. The existence of these cis-acting elements is only a preliminary prediction of gene function, and further experimental research is necessary to determine whether the overexpression of glyoxalase genes can directly protect plants from pathogen attacks. The identification of numerous cis-acting elements in the promoter regions of *PbrGLY* genes suggests their involvement in response to various biotic and abiotic stresses. The expression analysis of *PbrGLY* in response to *B. dothidea* infection revealed differential expression patterns among subfamilies, indicating their potential roles in disease resistance. The upregulation of specific *PbrGLY* genes, such as *PbrGLYI-28*, upon infection suggests their direct involvement in the pear’s defense response.

Existing research has confirmed the important role of the glyoxalase system in resisting various biotic and abiotic stresses. Recently, the glyoxalase pathway in rice has been reported to enhance tolerance to salt, drought, and extreme temperatures, and to reduce the damage caused by sheath blight (*Rhizoctonia solani*) [33]. The strain PXO99 of *Xanthomonas oryzae* pv. oryzae (*Xoo*) triggers the activation of the *OsWRKY62.1* transcription factor, which in turn suppresses the expression of *OsGLY II* genes by binding directly to their promoter regions. This regulatory action leads to an excessive accumulation of MG within the plant cells. The accumulation of MG has been shown to have an inhibitory effect on the rice plant’s resistance to the pathogenic strain PXO99 [23]. In grape, the transcription factor VvNAC72 directly interacts with the promoter of *VvGLYI-4*, thereby repressing its transcription. This downregulation of *VvGLYI-4* results in a reduction of its expression, concomitant with elevated levels of MG and reactive oxygen species (ROS), which correlates with enhanced resistance to downy mildew [5]. These results indicated that *GLY* genes played a key role in plant resistance to pathogen infection. However, it is still unclear whether glyoxalase genes are involved in the stress response to *B. dothide*a in pear. Through the expression analysis of *PbrGLY* after infection with *B. dothide*a, we found that most glyoxalase genes have high expression levels within 4 to 8 days. In particular, *PbrGLYI-28* has high expression levels 4 dpi, suggesting that it may play an important role in the defense response of pears to *B. dothide*a. The enhanced susceptibility of pears to the ring rot fungus after the transient silencing of the *PbrGLYI-28* directly confirms the important role of *PbrGLY* in pear resistance to *B. dothide*a. In addition, the glyoxalase activity of TRV2-*PbrGLYI-28* plants, suggesting *PbrGLYI-28* plant a major role in pear glyoxalase system.

The increased MG levels suggest reduced glyoxalase activity in TRV2-*PbrGLYI-28* plants. Infection by pathogens leads to an accumulation of reactive oxygen species (ROS) and a subsequent induction of antioxidant enzyme activity. Compared to TRV2 plants, TRV2-*PbrGLYI-28* plants exhibited greater ROS accumulation alongside diminished antioxidant enzyme activity. Furthermore, the expression levels of pathogen-associated genes were lower in TRV2-*PbrGLYI-28* plants relative to TRV2 plants. These findings provide new insights into the function of *PbrGLY-28* in pear resistance to ring rot disease and offer potential molecular targets for future disease-resistant breeding.

## Conclusions

This study systematically identified and analyzed the *PbrGLY* gene family in pear (*Pyrus bretschneideri*), with the aim of elucidating its role in pear resistance to *B. dothidea*. Through homologous comparison and conserved domain analysis, a total of 57 *PbrGLY* genes were identified, which are unevenly distributed across the pear chromosomes. Phylogenetic analysis revealed that the *PbrGLY* family can be divided into three major subfamilies, each with a varying number of members. Gene and protein structure analyses indicated that *PbrGLY* genes possess different numbers of exons and conserved motifs, and their promoter regions contain a variety of stress-responsive and hormone-responsive elements. qRT-PCR analysis showed significant changes in the expression levels of *PbrGLY* genes following infection by *B. dothidea*. Notably, the transient silencing of the *PbrGLYI-28* gene increased the susceptibility of pear to *B. dothidea*, elevated the content of methylglyoxal (MG), and reduced GLY activity. This study speculates that the *PbrGLY* gene family may have undergone functional differentiation and modulate the resistance of pear to *B. dothidea*. The expression variation of *PbrGLYI-28* may be associated with *B. dothidea* infection and the resistance of pear. These findings provide new insights into the molecular mechanisms underlying pear resistance to brown rot and offer a theoretical foundation and potential target genes for improving pear disease resistance through gene editing technologies. Future research will further verify the specific functions of these genes and explore their application potential in pear disease resistance breeding.

## Electronic supplementary material

Below is the link to the electronic supplementary material.

Supplementary Material 1

Supplementary Material 2

## Data Availability

The datasets supporting the conclusions of this article are included within the article and additional files.

## Abbreviations

CDS: Coding sequence
Dpi: Days post inoculation
GLY: Glyoxalase
HMM: Hidden Markov Model
UTRs: Untranslated regions
MG: Methylglyoxal
ROS: Reactive oxygen species
WGD: Whole genome duplication
VIGS: Virus-induced gene silencing
