# Supplementary material for: Genome-wide identification of glyoxalase (PbrGLY) gene family and functional analysis of PbrGLYI-28 in response to Botryosphaeria dothidea in pear
Source: BMC Plant Biol. 2025 Mar 18;25:349. doi: 10.1186/s12870-025-06302-6 (PMC11917052; doi:10.1186/s12870-025-06302-6)
Supplement: Supplementary file 1 — Supplementary Material 1 [file 12870_2025_6302_MOESM1_ESM.docx]

qRT-PCR Primers

| Primer name | Sequence |
| --- | --- |
| PbrGLYI-1F | CTGATTCCTAAGTTTTGTG |
| PbrGLYI-1R | TCTCAATCCAATAACCATC |
| PbrGLYI-2F | CTCCGAGTCCAGACCAGAT |
| PbrGLYI-2R | ACAGCCGAGTAGACAACAA |
| PbrGLYI-3F | AAATAAATAAAGATCCTGAGG |
| PbrGLYI-3R | TTGTGAGTGAGAAGGTGAG |
| PbrGLYI-4F | TTGGGGTTGTGGGGATGTT |
| PbrGLYI-4R | CACTAATCAAATTACGCATAT |
| PbrGLYI-5F | CAATTTGCAGGTCTTTGTT |
| PbrGLYI-5R | AGCGTTAGGAGGTTAGGAT |
| PbrGLYI-6F | CCGCCAAGAAGGATGAGAA |
| PbrGLYI-6R | AAGCGAGAAAGAACGAAGGAC |
| PbrGLYI-7F | AGGGCGCAGAGGAAGGTGA |
| PbrGLYI-7R | GCCAATCCGATAGCCGTAG |
| PbrGLYI-8F | AAATAGTATCGCGAACTTT |
| PbrGLYI-8R | TTTTCGAGTGTTCTGACTG |
| PbrGLYI-9F | GTAAAGACGTGGATGGAGA |
| PbrGLYI-9R | AGAGCAATAGCCGCGAAAT |
| PbrGLYI-10F | GACATAAAGCATCGCGGGTAG |
| PbrGLYI-10R | CGTCAGTGGCTGAGGAAGA |
| PbrGLYI-11F | TTGGGTCTTGGCGTTTAGT |
| PbrGLYI-11R | GGATCGCTTGTATTTCAGT |
| PbrGLYI-12F | AACTGTTGGATGTGGGATT |
| PbrGLYI-12R | TTCGGTGGTGCTGTGGAAT |
| PbrGLYI-13F | GTTTTGTGCTCTATGTATT |
| PbrGLYI-13R | CAGCTACTAAAATAACCAT |
| PbrGLYI-14F | ACATTTCTTTCCAGGTCTC |
| PbrGLYI-14R | GATCAACCGTCTAACTACA |
| PbrGLYI-15F | CCACCGCTTCTTGTCTACTGG |
| PbrGLYI-15R | GAGATTCAAGTATATTCACACACAG |
| PbrGLYI-16F | CCACCGCTTCTTGTCTACTGG |
| PbrGLYI-16R | ACGCAATCTTCCTCGCATT |
| PbrGLYI-17F | AAAACGGAACCACAATCGA |
| PbrGLYI-17R | GGCGGAGTATGCCTATCAA |
| PbrGLYI-18F | TTAGTCTTTCGATCCGGTGAC |
| PbrGLYI-18R | TGAGCGTTGCTGCTTCTCC |
| PbrGLYI-19F | GCAGGAAGATGGGATTAGA |
| PbrGLYI-19R | CTTAGCTTTCGCCACAGAA |
| PbrGLYI-20F | CTTACGAACCCATCGTCTA |
| PbrGLYI-20R | TGTCCTGAGCACCAACTGA |
| PbrGLYI-21F | TCTTAGGCTAGATGAGGCA |
| PbrGLYI-21R | TAGACCGTATAAGGGAAGT |
| Primer name | Sequence |
| PbrGLYI-22F | TAATCAAACGGTGACCAAGT |
| PbrGLYI-22R | CTAAGCAGGTTCATAGAGGC |
| PbrGLYI-23F | CTGTTTCTCACTGGGTTTT |
| PbrGLYI-23R | TAGCAATTTAGCATCTCCC |
| PbrGLYI-24F | AATTGGGTTTGAGCAGAGG |
| PbrGLYI-24R | TGTTGGTGGATTGACATAG |
| PbrGLYI-25F | ACCGCCTTCTACTCCAAAG |
| PbrGLYI-25R | TGTGCAAATTCATTCCCTA |
| PbrGLYI-26F | TTGCCAGTCACTTAATCTG |
| PbrGLYI-26R | CAAACCTCCTTGACCCAAT |
| PbrGLYI-27F | TGTTACTGTTGGTGCCTATT |
| PbrGLYI-27R | CACATTACAAGCACGTTGA |
| PbrGLYI-28F | CGCCATCAAGTTCTACGAG |
| PbrGLYI-28R | GAAGGTGAAGGACCCAAGC |
| PbrGLYII-1F | GCACAGCAGCAGCGTCAAG |
| PbrGLYII-1R | GGTAGATCGGCACGGATAA |
| PbrGLYII-2F | TTCCGATTCCCGAACGATG |
| PbrGLYII-2R | GGCGAGCAAGTAAGTGTAGGT |
| PbrGLYII-3F | ATGCTGTTCTTTGCTTTTC |
| PbrGLYII-3R | CATTTTCTTGATTACCCTTT |
| PbrGLYII-4F | CAGGTTCTCCGCAAAGGTA |
| PbrGLYII-4R | CGGGACGATAATGCAGGTC |
| PbrGLYII-5F | TCATGTTGGTGGCTGTGCT |
| PbrGLYII-5R | GCGATCTGGCGTAAAGTCC |
| PbrGLYII-6F | GATTAAATCTGACGGTGGT |
| PbrGLYII-6R | TAAGAGGGTAAGGAGACGA |
| PbrGLYII-7F | CAGTGGATACGGAGGATAG |
| PbrGLYII-7R | GAGTACCGACCTCAATAGC |
| PbrGLYII-8F | ACCGGAAGCTCGCTGTGAT |
| PbrGLYII-8R | ATTGGGAGGAGGGCAAGTC |
| PbrGLYII-9F | CAATAGAAATGCCGAAAGG |
| PbrGLYII-9R | ATTGGGTAAGAGGAGTGGG |
| PbrGLYII-10F | CTCTACCCGGCTCCGTTCA |
| PbrGLYII-10R | CCGCCACAAGAGGCTGTAA |
| PbrGLYII-11F | TGTGAAGTAGGCTAGGTTT |
| PbrGLYII-11R | AGTCATACTAGGCAGAAATA |
| PbrGLYII-12F | TTAATCAAGCCTCGACCCG |
| PbrGLYII-12R | TTCCATTGACGCCAACACT |
| PbrGLYII-13F | ATGGCTTCAATGGGACAAC |
| PbrGLYII-13R | AGCACCGAGTGGAGTAATG |
| PbrGLYII-14F | TGGAACAAGTAGGGTCTAAG |
| PbrGLYII-14R | ATAAACACCGAATTGATGG |
| PbrGLYII-15F | CCTCCTGTAATCCGCACCA |
| Primer name | Sequence |
| PbrGLYII-15R | GAGACATCGGCGAGCAAGT |
| PbrGLYII-16F | AATGCGGTGTTTGCAGGGTA |
| PbrGLYII-16R | TCGGTGGAGAAGACGGGAG |
| PbrGLYII-17F | GGGGCACAGTGATGAATAT |
| PbrGLYII-17R | CAATCCACAAACGGAGAAC |
| PbrGLYII-18F | GCTAAATTCCGCTCCTTCT |
| PbrGLYII-18R | TTTGTAACAATCGCAACCT |
| PbrGLYII-19F | TGTCCGAGAAGTTAATGAG |
| PbrGLYII-19R | ATGGTAAGGACGATGTGAT |
| PbrGLYII-20F | AATCTGCTCCATACATTCA |
| PbrGLYII-20R | CAAATAGGGATCACTTTCA |
| PbrGLYII-21F | TCCCATATTCATTTCTCCG |
| PbrGLYII-21R | GACAAGGGTTTCGGTCTTT |
| PbrGLYIII-1F | TAAGTTGCATCCCCATACT |
| PbrGLYIII-1R | CAAACTCCGTAAAGCACAA |
| PbrGLYIII-2F | CACATAGCAGGCACTCCAT |
| PbrGLYIII-2R | AAGCCTTCTCAGAGCTACAAC |
| PbrGLYIII-3F | CCATTTAGGATTAGGGTGT |
| PbrGLYIII-3R | ACTAGAACTTCACGGTGGT |
| PbrGLYIII-4F | GTGGTTCTTTCGGGTGGCA |
| PbrGLYIII-4R | GAGAAATGAACTCTGGGTG |
| PbrGLYIII-5F | ATTGGGTGGAGGTGGTTAT |
| PbrGLYIII-5R | CAAGGGTAGCAGGTGAAGC |
| PbrGLYIII-6F | GCTGAAGGGACTGAAGGTA |
| PbrGLYIII-6R | AAGACAATAGAGCCCACAA |
| PbrGLYIII-7F | CAGAGGACTTTCTACCGTCAC |
| PbrGLYIII-7R | GGGTAATCATAAACGAGGC |
| PbrGLYIII-8F | CAACCAAACGGAACTTTAA |
| PbrGLYIII-8R | TTTCTTCTGTCCCATACCC |
| PbNPR1-F  PbNPR1-R  PbPR1-F | AGAGGGTAAAGAGGAGGCGT  AGGCTCAACTCAGCCTTGTC  AACAACGAGACCGTCTGGAG |
| PbPR1-R | TCGGATTTTCTCTGGCCTGC |
| PbPR5-F | ACGACGCAGGAAATGGAAA |
| PbPR5-R | TTGGACCCGATTGTGAACTC |
| Tubulin-F | TGGGCTTTGCTCCTCTTAC |
| Tubulin-R | CCTTCGTGCTCATCTTACC |

Note: The PbrGLY, PbrNPR1, PbrPR1 and PbrPR5 primers were employed in the qRT-PCR experiments, with Tubulin serving as the reference gene.

Gene Primers

| Primer name | Sequence |
| --- | --- |
| pTRV2-PbrGLYI-28F： | gtgagtaaggttaccgaattcATGGCGGAGCAAAAGGTACA |
| pTRV2-PbrGLYI-28R： | cgtgagctcggtaccggatccCAGACTCAGTCACAAGGAAGGTGA |

Note: The aforementioned primers were utilized in the construction of the *pTRV2-PbrGLYI-28* silencing vector for the VIGS experiment. The lowercase letters denote the homologous arms of the constructed vector.
