# Supplementary material for: Genome-wide identification of glyoxalase (PbrGLY) gene family and functional analysis of PbrGLYI-28 in response to Botryosphaeria dothidea in pear
Source: BMC Plant Biol. 2025 Mar 18;25:349. doi: 10.1186/s12870-025-06302-6 (PMC11917052; doi:10.1186/s12870-025-06302-6)
Supplement: Supplementary file 2 — Supplementary Material 2 [file 12870_2025_6302_MOESM2_ESM.docx]

Table S2. Motif Information Summary

| Motif Number | Logo | Sites | Width | E-value |
| --- | --- | --- | --- | --- |
| Motif 1 | 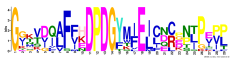 | 15 | 29 | 1.40E-148 |
| Motif 2 | 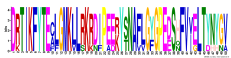 | 6 | 50 | 4E-146 |
| Motif 3 | 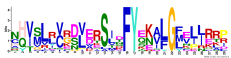 | 18 | 29 | 7.1E-134 |
| Motif 4 | 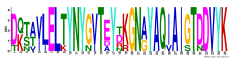 | 6 | 35 | 4.4E-94 |
| Motif 5 | 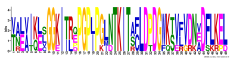 | 6 | 49 | 2.2E-98 |
| Motif 6 | 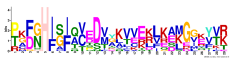 | 17 | 29 | 2.4E-114 |
| Motif 7 | 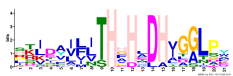 | 21 | 21 | 1.2E-93 |
| Motif 8 | 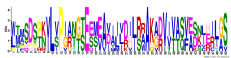 | 8 | 50 | 3.5E-93 |
| Motif 9 | 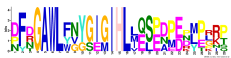 | 8 | 29 | 1.1E-73 |
| Motif 10 | 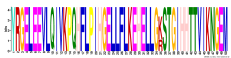 | 3 | 50 | 5E-49 |
